# Symbiont-Targeted Control of *Halyomorpha halys* Does Not Affect Local Insect Diversity in a Hazelnut Orchard

**DOI:** 10.3390/insects16070688

**Published:** 2025-06-30

**Authors:** Sofia Victoria Prieto, Matteo Dho, Bianca Orrù, Elena Gonella, Alberto Alma

**Affiliations:** 1 Department of Agricultural, Forest and Food Sciences, University of Torino, 10095 Grugliasco, Italyelena.gonella@unito.it (E.G.);; 2 Interdepartmental Centre for Innovation in the Agro-Environmental Sector, AGROINNOVA, University of Torino, 10095 Grugliasco, Italy

**Keywords:** non-target insects, alpha diversity, brown marmorated stink bug, kernel damage, *Corylus avellana*, orchard

## Abstract

Harmless crop-associated insect communities are a fundamental part of the agroecosystem. Their potential as a reservoir of natural enemies of pests has encouraged their conservation through the development of low-impact pest management programs. The brown marmorated stink bug, *Halyomorpha halys,* represents a serious threat to Italian hazelnut production. Laboratory and field experiments confirmed the susceptibility of this pest to the disruption of the obligated symbiotic interaction with gut bacteria, paving the way for the development of the symbiont-targeted control strategy. Here we present the results of a three-year field assessment of symbiont-targeted control in a hazelnut orchard in northwestern Italy. The use of a biocomplex to disrupt symbiont acquisition by *H. halys* nymphs was compared to the use of lambda-cyhalothrin insecticide. The effects on the local entomofauna were assessed, as were the trend of *H. halys* population and the damage caused by stink bugs to harvested hazelnuts. The insecticide consistently reduced the insect diversity in the field, while the anti-symbiont biocomplex had no effect. However, the control of the *H. halys* population and the stink bug-induced damage to hazelnuts varied over the years in the field plot submitted to the symbiont-targeted approach. Our results indicate that the symbiont-targeted control does not interfere with local insect communities. Key aspects for improving the effectiveness of this tactic are discussed.

## Simple Summary

*Halyomorpha halys* is an invasive stink bug that affects hazelnut production in Italy. A recently developed approach to control this pest is the symbiotic control conducted by interrupting the positive interactions established between *H. halys* and bacteria (i.e., symbiont-targeted control). In here, the symbiotic control was compared to insecticide use in a hazelnut orchard, taking into account the effects on non-target insects and on *H. halys* population. Damage associated with stink bugs was also evaluated on post-harvest hazelnuts. The symbiont-targeted control did not affect the insect diversity, whereas the use of lambda-cyhalothrin significantly reduced it. The impact of the symbiont-targeted control on the trend of *H. halys* population varied over the years, as did the intensity of damage to hazelnuts. Our field experiments confirmed the lack of interference of symbiont-targeted control with the local insect community, while several agronomic aspects are discussed here to improve the efficacy of this strategy.

## 1. Introduction

Non-pest insects are cornerstones of crop production, not only as pollinators but also for other supporting and regulating services, including those provided by predators and parasitoids of crop-feeding pests [1,2]. Recently, the beneficial services provided by insects to agriculture have become increasingly important, especially since the implementation of the Integrated Pest Management (IPM) [1]. In this context, the conservation of entomological biodiversity in the crop environment allows for the growth of beneficial insect populations and acts as a reservoir of new predator or parasitoid species yet to be identified.

Italy is the second largest producer of hazelnuts in the world, after Turkey and followed by the United States of America, with a production of 102,740 tonnes [3]. A remarkable diversity of insect species is recorded in European hazelnut orchards, most of which do not pose a threat to the crop (i.e., less than 30% are considered pests) [4]. This diversity includes both variability of taxa and feeding behaviours [5]. The need to reduce the use of insecticides in hazelnut orchards has led to the incorporation of new approaches to pest management, many of which are based on the use of antagonistic species (i.e., biological control) [6]. For example, in Oregon (USA), the introduction of the parasitoid *Trioxys palludis* (Halliday) (Hymenoptera: Braconidae) against filbert aphids dramatically reduced the use of insecticides [7]. In this way, successful biological control ultimately enhances the survival of other beneficial insects, which can potentially contrast other pests.

Since it was first recorded in Italy in 2007, the brown marmorated stink bug *Halyomorpha halys* (Stål) (Hemiptera: Pentatomidae) has caused increasing damage to several crops, including hazelnuts [8,9]. This invasive pest, native to eastern Asia and highly polyphagous, has spread to North America and Europe over the last 20 years and has recently been recorded in South America and North Africa [10,11]. Other countries expect its arrival in the coming years, as it has already been detected at borders associated with the international transport of goods [12].

The damage caused by *H. halys* to hazelnut is related to the phenological phase of the crop in which the injury occurs and can range from seed abortion to visually detectable symptoms on the kernels that lower the quality of the fruit [13]. Additionally, the injection of digestive enzymes as a consequence of the insect feeding behaviour affects the kernel composition (e.g., degrading proteins due to the activity of protease enzymes), harming the commercialisation of hazelnuts [14].

Traditionally, pest management of *H. halys* relied on broad-spectrum synthetic insecticides, which are mostly incompatible with IPM [15]. The drawbacks of insecticides (e.g., environmental and public health hazards, water and soil contamination and impact on non-target species) led to a phase-out of many active substances in Europe [16]. As a result, there has been a strong push in recent years for different pest management strategies that do not rely exclusively on the use of insecticides [17]. For example, biological control based on the use of Hymenoptera egg parasitoids against *H. halys* is currently active in hazelnut orchards in northwestern (NW) Italy [18,19]. Augmentative biological control with native egg parasitoids (e.g., *Anastatus bifasciatus* (Geoffroy) (Hymenoptera: Eupelmidae)) is being enhanced by the release of the exotic wasp *Trissolcus japonicus* (Ashmead) (Hymenoptera: Scelionidae), a natural enemy of *H. halys* in its native range [20].

Harnessing the interaction between insects and microorganisms is a promising area for the development of pest management strategies [21]. Pentatomids are involved in symbiotic interactions with *Pantoea* spp., transmitted vertically to the progeny through egg smearing and acquired orally after hatching [22,23]. Treatment of egg masses with bactericidal substances prevents symbiont acquisition [24]. In *H. halys*, the interruption of symbiont transmission leads to early nymphal mortality and imperfect development [25,26,27,28]. Symbiont-targeted control aims at disrupting symbiont acquisition by treating egg masses with low-impact substances compatible with IPM [29].

In here, we present the results of a three-year field trial in a hazelnut orchard in NW Italy to evaluate the effect of symbiont-targeted control on non-target species. A biocomplex previously tested under laboratory and field conditions was used to eliminate symbiotic bacteria from the egg surface [26,30,31]. Periodic surveys were performed after treatment to assess the trend of *H. halys* population; additionally, the abundance of other insect families was estimated to consider potential alterations of the local fauna. The effectiveness of the anti-symbiont biocomplex against the target insect was compared to that of an insecticide, as well as compatibility with the conservation of insect diversity.

## 2. Materials and Methods

### 2.1. Symbiont-Targeted Control Field Treatments

Field trials were performed in a commercial hazelnut orchard (cultivar Tonda Gentile delle Langhe) located in the municipality of Barge (Piedmont, Italy; 44°44′59.1″ N, 7°25′28.0″ E) during June and July 2022–2024 (Table 1). The orchard was situated in a heterogeneous landscape surrounded by different crops such as corn and blueberries. The borders of the orchard contained spontaneous plant species (mainly *Aegopodium podagraria*, *Phytolacca* spp. and *Fraxinus* spp.). Within the orchard, three one-hectare plots were randomly selected, and each was randomly assigned to a treatment: (1) application of a micronutrient biocomplex (Dentamet^®^—Diachem S.p.A., Caravaggio, Italy), previously used for anti-symbiont egg mass treatment, at a dose of 0.3 L/hL [26,30]; (2) treatment with lambda-cyhalothrin (Atlas^®^—Ascenza Italia S.r.l, Saronno, Italy), an insecticide commonly used in hazelnut production, at a dose of 0.2 L/hL; (3) untreated control, where no insect management was conducted. All other farming practices (e.g., fungicide treatments) were the same in all plots. To determine the beginning of the anti-symbiont treatment, a pre-treatment survey (i.e., survey zero) was performed and the number of egg masses and individuals of *H. halys* was recorded. Visual inspections during survey zero consisted in randomly choosing 20 hazelnut trees distributed throughout the orchard and spending five minutes per plant in search of egg masses in the leaves. The trials lasted four weeks, starting in late June: four applications of the anti-symbiont biocomplex were made every 10 days (±1) starting from the first report of *H. halys* egg masses in the field until nut drop. In 2022, due to the late detection of *H. halys* egg masses at the beginning of the trial, only 3 treatments were applied. In the dedicated plot, an insecticide treatment was carried out after the first observation of adult *H. halys*, followed by a second treatment after two weeks (Table 1).

### 2.2. Assessment of Insect Community Composition

Insect surveys were conducted every 9 days (±3) after the symbiont-targeted treatment (Table 1). The first survey of 2022 assessed only the effect of the insecticide, as no biocomplex application had been applied at that date.

For the insect sampling, each plot was divided into three sub-plots (approximately 0.3 hectares each), and three hazelnut trees were randomly selected within each sub-plot for both visual monitoring (targeting stink bug egg masses and any other immobile insect stages) and beat sheet sampling. Egg masses were collected and stored in the laboratory for parasitism detection; in the event of parasitoid emergence, wasps were identified to the species level. Egg masses were classified as “fresh” (not yet hatched), “hatched with nymphs” (if first/second instar nymphs were observed together surrounding the hatched egg mass), “hatched” (no record of nymphs surrounding the egg mass) and “parasitised” (if egg parasitoid emergence was recorded later in the laboratory). The beat sheet sampling consisted in scouting six (±1) branches of each selected tree over a white nylon tarpaulin (1.0 × 1.0 m) placed on the ground and collecting the fallen insects. The collected individuals were preserved in 70% ethanol in 50 mL tubes and subsequently identified at the family level by Enrico Busato (Department of Agricultural, Forest and Food Sciences, University of Torino). Egg parasitoids that emerged from field-collected Pentatomidae egg masses were identified by Francesco Tortorici (Department of Agricultural, Forest and Food Sciences, University of Torino). A stereomicroscope SZH-10 (Olympus, Tokyo, Japan) was used for the identification of small specimens. In the case of Pentatomidae, an additional identification at the subfamily level was conducted to discriminate between plant-feeding and zoophytophagous species [32]. Additionally, the main pentatomid pests of hazelnut in NW Italy (i.e., *H. halys*, *Palomena prasina* Linnaeus and *Nezara viridula* Linnaeus [13]) were identified at the species level.

### 2.3. Damage Evaluation on Hazelnuts

After harvesting, a total of 400 hazelnuts per treatment were separately preserved. In the laboratory, the hazelnuts were shelled and visually inspected for symptoms associated with stink bug damage. Each kernel was first externally observed and classified as: “damaged”, if stink bug-related symptoms were observed, such as white or brown tissue (or both); “healthy”, with no visible damage symptoms; and “other”, a third category that included damage not directly attributable to stink bug feeding, such as mouldy, shrivelled and blank nuts (the latter related to the hazelnut and detected during shelling) [13,33].

### 2.4. Statistical Analysis

Alpha diversity, measured by the Shannon index, was evaluated to assess the potential impact of the treatments on the orchard entomofauna. Statistical differences in the alpha diversity among treatments were evaluated using a one-way ANOVA test after confirming normal distribution and variance homogeneity with the Shapiro-Wilk normality test and Levene’s test, respectively. Means were separated using Bonferroni’s post-hoc test. The same approach was used to detect differences in the number of adults and nymphs recorded per survey and treatment. The abundance per insect family was normalized by applying the square root to the number of individuals recorded per family per sampling unit. Kernel damage, expressed as proportions relative to the total number of hazelnuts evaluated, was statistically evaluated using a binomial GLM model with a logit link function, and means were separated using Bonferroni’s post-hoc test.

## 3. Results

### 3.1. Diversity of Entomofauna Recorded in Field

**Figure 1:**
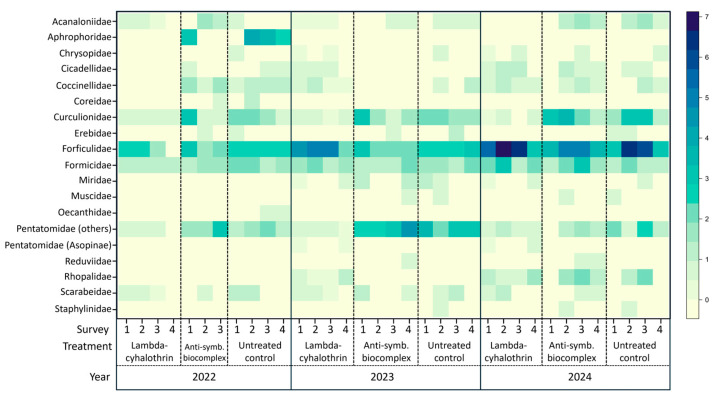
Heat map indicating the abundance of insects (identified at the family level) recorded in field throughout the three-years trial, according to the treatment.

Regarding the taxonomic affiliation of the collected insects, a total of 19 families belonging to 10 orders was recorded (Figure 1, Table S1). The most abundant families were Forficulidae, Formicidae, Curculionidae and Pentatomidae. For the two most abundant groups (Forficulidae and Formicidae), the abundance remained relatively constant for all three years and treatments, although an increase in the presence of Forficulidae was observed all throughout the orchard in year 2024. The main difference observed among treatments was the reduction in the abundance (or complete absence) of certain families in the lambda-cyhalothrin treated plot. This effect was observed for groups such as Pentatomidae, Curculionidae and Coccinellidae (the latter mainly in 2022 and 2023). In all three years, most of the Pentatomidae corresponded to plant-feeding species (mainly *H. halys*), while the zoophytophagous Asopinae species were rarely present (i.e., only the species *Arma kustos* Fabricius was recorded).

**Figure 2:**
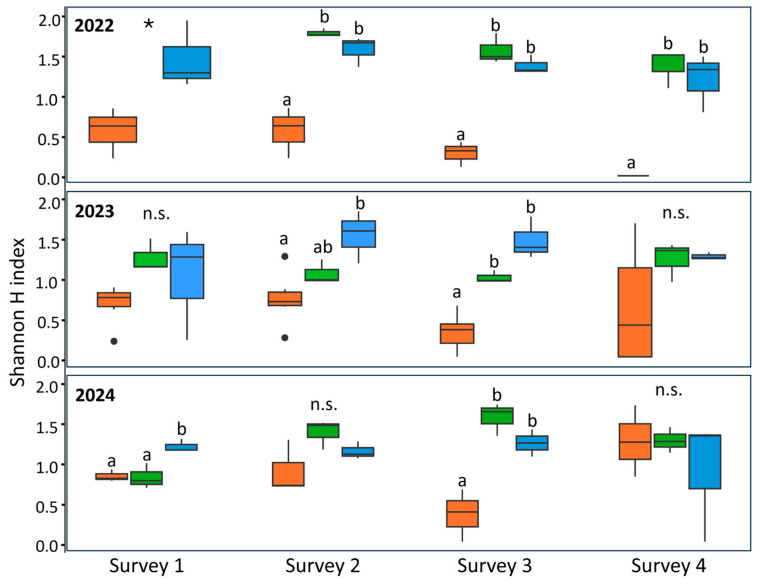
Alpha diversity measured by the Shannon H index recorded in the three years surveyed, according to the treatment. Orange: lambda-cyhalothrin; green: anti-symbiont biocomplex; blue: untreated control. Different letters indicate significant differences among adjacent bars according to one-way ANOVA followed by Bonferroni’s post-hoc test. Asterisks indicate significant differences between adjacent bars. Black dots represent outliers. n.s. stands for no significant differences.

The diversity of insects collected through the beat sheet technique was evaluated using the Shannon H index of alpha diversity (Figure 2, Table S1). In 2022, the plot treated with lambda-cyhalothrin had a lower insect diversity than the other plots in all surveys performed (one-way ANOVA: survey one: df = 1, *F* = 8.59, *p* value = 0.043; survey two: df = 2, *F* = 27.67, *p* value < 0.01; survey three: df = 2, *F* = 58.36, *p* value < 0.01; survey four: df = 2, *F* = 26.7, *p* value = 0.001) (Figure 2). According to Bonferroni’s post-hoc tests, alpha diversity was equal in the anti-symbiont biocomplex and the untreated plots. In 2023, no differences in the alpha diversity were observed in surveys one and four (one-way ANOVA: survey one: df = 2, *F* = 2.38, *p* value = 0.148; survey four: df = 2, *F* = 1.89, *p* value = 0.206) (Figure 2). Differences were recorded in survey two, with the lowest alpha diversity observed in the insecticide-treated plot and the highest in the untreated plot, with the biocomplex-treated plot representing an intermediate condition (one-way ANOVA, df = 2, *F* = 7.11, *p* value = 0.014) (Figure 2). Finally, a similar pattern to the previous year was observed in survey three, with the lambda-cyhalothrin treated plot having significantly lower diversity compared to the other two treatments (one-way ANOVA, df = 2, *F* = 31.2, *p* value < 0.01) (Figure 2). In 2024, no differences in alpha diversity among treatments were observed in surveys two and four (one-way ANOVA; survey two: df = 2, *F* = 3.24, *p* value = 0.11; survey four: df = 2, *F* = 0.50, *p* value = 0.63). However, in survey one, the alpha diversity was lower in both the insecticide and the anti-symbiont biocomplex plots compared to the untreated area (one-way ANOVA, df = 2, *F* = 11.39, *p* value = 0.009). Finally, in survey three, diversity was lower in the lambda-cyhalothrin-treated plot compared to the other two treatments, for which no differences were recorded (one-way ANOVA, df = 2, *F* = 10.3, *p* value = 0.01).

### 3.2. Trend of Pentatomid Pest Populations

**Figure 3:**
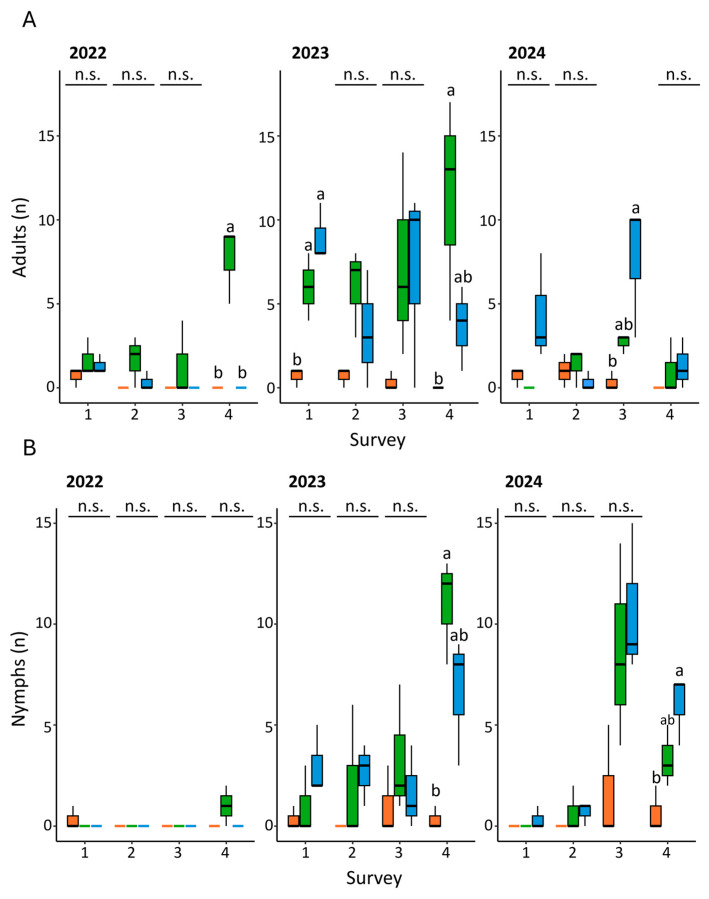
Trend of adults (**A**) and nymphs (**B**) of *H. halys* recorded in field according to the year, treatment and survey. Orange: lambda-cyhalothrin; green: anti-symbiont biocomplex; blue: untreated control. Different letters indicate significant differences among adjacent bars according to one-way ANOVA followed by Bonferroni’s post-hoc test. n.s. stands for no significant differences.

In general, both adults and nymphs of *H. halys* were less present in the field area treated with lambda-cyhalothrin (Figure 3A,B, Table S2). As to the adults, at the beginning of the trial (survey zero), the number of individuals recorded in the field varied from one to three. Statistical differences were detected only in surveys four (2022), one and four (2023) and three (2024) (Figure 3A, Table S2). In all cases the lowest records corresponded to the lambda-cyhalothrin treatment, while the anti-symbiont biocomplex showed a fluctuating impact on the trend of adults (Figure 3A, Table S2).

As to the nymphs, no significant differences were recorded among treatments and surveys, except for the last two surveys of 2023 and 2024 (Figure 3B, Table S2). In both cases, the number of nymphs recorded in the plots treated with lambda-cyhalothrin was lower when compared to the anti-symbiont complex and the untreated control. The trials performed during 2022 displayed the lowest levels of infestation regardless of treatment (Figure 3A,B).

The presence of *H. halys* egg masses was also recorded throughout the surveys. Fresh egg masses were found during survey zero only in 2023 and 2024 (one egg mass each year, respectively). In 2022, the first fresh egg mass of *H. halys* was detected during survey one. After that, a total of five egg masses were found, all of them in the biocomplex treated plot: one fresh (survey two), one fresh and one hatched (survey three) and two hatched with nymphs (survey four). In 2023, three egg masses were sampled in the anti-symbiont biocomplex plot (two hatched with nymphs, survey one; one parasitised, survey three) and one hatched with nymphs in the control plot, survey one. The parasitoids emerging from the parasitised egg masses were all identified as *T. japonicus*. Finally, ten egg masses were counted in 2024. Two of these were in the untreated control plot (one fresh, survey two; one hatched with nymphs, survey three); one in the insecticide-treated plot (hatched, survey three); and the rest in the biocomplex-treated plot (one fresh, survey two; one hatched and three parasitised, survey three; one hatched with nymphs, one hatched and one parasitised, survey four). Regarding the parasitised egg masses, the parasitoids emerging from the three egg masses parasitised recorded during survey three belonged to the species *Trissolcus mitsukurii* (Ashmead) (Hymenoptera: Scelionidae), while the egg mass recorded in survey four was parasitised by *T. japonicus*.

In comparison to *H. halys*, a few other pest pentatomids were sampled in the field in all three years, all in the adult stage. In 2022, six adults of other phytophagous stink bugs were recorded (all in the anti-symbiont biocomplex plot): one *P. prasina* (survey two), one *Dolycoris baccarum* Linnaeus (survey three) and five *N. viridula* (survey four). In 2023, only two *N. viridula* individuals were detected (survey two, anti-symbiont biocomplex plot; survey four, untreated control, respectively). Finally, in 2024, other pentatomids were only observed during survey four (one *N. viridula* adult in the anti-symbiont treated plot and one *D. baccarum* adult in the untreated plot).

### 3.3. Stink Bug—Related Damage

**Figure 4:**
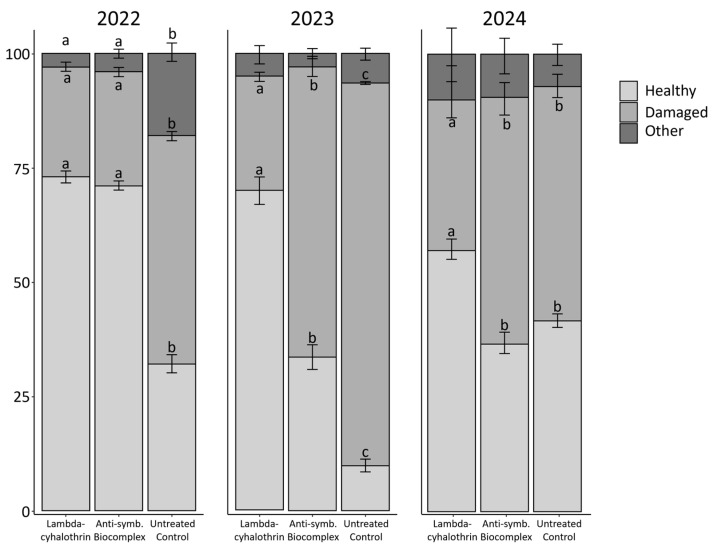
Damage percentage recorded in hazelnuts according to the year and the treatment. Different letters indicate significant differences between adjacent bars with the same colour, according to binomial GLM with Bonferroni’s post-hoc test. No letters indicate no significant differences.

In 2022, the level of damage caused by stink bugs in kernels varied according to the treatment (binomial GLM, df = 2, χ^2^ = 192.4, *p* value < 0.01) (Figure 4). Bonferroni’s post-hoc tests indicated that the lowest level of damage was present in the kernels derived from the lambda-cyhalothrin and the anti-symbiont biocomplex plots (statistically equal), while the highest level of damage was detected in the untreated control. The same pattern was observed in the categories “healthy” and “other” for the same year (binomial GLM; healthy: df = 2, χ^2^ = 433.6, *p* value < 0.01; other: df = 2, χ^2^ = 168.3, *p* value < 0.01) (Figure 4). In 2023, the highest level of stink bug damage in kernels was observed for the untreated control, followed by the anti-symbiont biocomplex and lambda-cyhalothrin (binomial GLM; df = 2, χ^2^ = 492.06, *p* value < 0.01) (Figure 4). Bonferroni’s post-hoc test showed an intermediate level of damage in the hazelnuts from the anti-symbiont treatment, and the same pattern was observed for the level of healthy kernels. No differences were found for other types of damage this year (binomial GLM; df = 2, χ^2^ = 5.93, *p* value = 0.052) (Figure 4). In 2024, the level of damage recorded for the insecticide treatment increased compared to that of the previous years. Statistical differences among treatments were recorded when comparing the level of damaged and healthy kernels (binomial GLM; damaged: df = 2, χ^2^ = 49.7, *p* value < 0.01; healthy: df = 2, χ^2^ = 24.7, *p* value < 0.01). Nonetheless, the lambda-cyhalothrin treatment represented the lowest level of damage to hazelnuts, while no differences were found between the biocomplex and the control according to Bonferroni’s post-hoc test. Also in this year, the “other” damage category was equal among treatments (binomial GLM, df = 2, χ^2^ = 2.5, *p* value = 0.28). A comparison of the damage attributed to stink bugs was performed among years, which confirmed different levels of damaged hazelnuts: the lowest levels were observed during 2022, while the highest were detected during 2023 (binomial GLM, df = 2, χ^2^ = 385.14, *p* value < 0.01). The year 2024 corresponded to an intermediate condition.

## 4. Discussion

The implementation of a new pest management approach faces multi-factorial challenges, including the difficulty of reproducing laboratory successful strategies in the field. With the increasing incorporation of biological control agents into IPM, ensuring a non-interference between a new pest control tactic and non-target insects becomes essential. In here, the efficacy of a symbiont-targeted control approach against *H. halys* was tested in a hazelnut orchard, based on the results previously obtained under controlled conditions. Our experiments focused not only on tracking the effect on the target pest population but especially on the potential side effects on the co-existing insect community.

In terms of the alpha diversity of the insect community in the orchard, a consistent decrease was observed in the plot treated with lambda-cyhalothrin. This event was more pronounced in 2022, where all surveys showed the same pattern. The detrimental effect of insecticides on the biodiversity has been well documented [34,35,36]. However, insect families were not equally affected by the insecticide treatment. The most reduced groups were Pentatomidae, Curculionidae, Coccinellidae and Acanaloniidae. The diminution in the Pentatomidae family (i.e., the treatment target) agrees with the reduction in the number of adults and nymphs and in the stink bug-associated damage in kernels from the plot treated with lambda-cyhalothrin in all three years. The recorded specimens belonging to the family Curculionidae were mostly *Phyllobius* spp., a minor hazelnut leaf-feeder [37]. Other groups that were affected by lambda-cyhalothrin such as Coccinellidae are widespread natural enemies of insect pests [38]; therefore, their reduction may favour the rise of populations of secondary pests such as scale insects or eryophid mites. On the contrary, no effect on insect diversity was inferred from the use of the anti-symbiont biocomplex, as the alpha diversity in the plot corresponding to this treatment was mostly equal to that in the control plot. The lack of impact on the co-occurring entomofauna is a decisive aspect when developing a containment strategy against an insect pest. In an integrated approach to pest management, the absence of interference between the symbiotic control and biological control agents could be expected. Furthermore, the latter has been previously confirmed in laboratory assessments with *H. halys* egg parasitoids [39]. The retrieval of egg masses that had been successfully parasitised by *Trissolcus* spp. in the biocomplex-treated plot provides a field confirmation of the compatibility of these two strategies.

The effect of symbiont-targeted control on *H. halys* varied over the years. During 2022, the level of infestation was lower compared to the other two years, probably due to an increase in the mean temperature [40] that may have negatively affected the fitness of *H. halys* population [41]. In general, the trend of adults displayed an unsteady pattern, with lower levels in the lambda-cyhalothrin-treated plots. The increase in the number of adults (that are not directly affected by the anti-symbiont treatment) may be related to the constant arrival of individuals from the surrounding areas. Indeed, a high number of adults of *H. halys* was detected in the wild plants around the orchard, such as *Fraxinus* spp. trees. Moreover, among the pentatomids sampled, *H. halys* was largely the most abundant. This pattern agrees with previous studies in different fruit crops in several Italian regions [9,42], although previous field surveys in hazelnut orchards in Piedmont indicated a more balanced situation, with other pentatomid species recorded at similar or higher levels than *H. halys* [13]. Nonetheless, high temporal and site-specific variability is expected between separate stink bug surveys, suggesting that different studies are difficult to compare.

For the most part, no differences were recorded throughout the treatments in the number of *H. halys* nymphs, except for survey four in 2023 and 2024. The lack of a consistent response to the symbiotic control in the juvenile individuals trend contrasts with what was observed previously in a field trial performed with the same biocomplex in a soybean crop [31], suggesting that variability in the treatment efficacy can be expected when applying it to different cultures. As for the egg masses, it should be noted that a low quantity was found during the surveys; therefore, the results concerning their presence and distribution only provide a qualitative confirmation of the infestation but do not allow quantifying the stink bug abundance.

Several agronomic factors related to the implementation of the symbiotic control in the field deserve further analysis. The canopy structure of the crop is a crucial aspect since the target is the egg mass; considering that stink bugs tend to lay eggs in the underside of the leaves [43], both species-specific (i.e., leaf disposition) and agronomic (i.e., crop training system, pruning practices) features need to be considered to improve efficacy. Optimisation of spray application was addressed by Dho et al. (2025) [30], whose results indicated a higher egg mass coverage in the central part of the canopy. Another critical point remains the timing of application, as the anti-symbiont biocomplex needs to be applied in conjunction with the presence of egg masses in the field. In this study the treatments with the anti-symbiont biocomplex started after the detection of at least one egg mass in the field; therefore, the application of thorough monitoring to detect the presence of egg masses in the field is recommended to maximise the efficacy of symbiont-targeted control. Once oviposition in the field has started, the risk of not completely covering the egg masses laid in the period between two consecutive treatments cannot be avoided even with frequent treatments. The choice to perform the treatments every ten days can be considered the best compromise between the ontogeny of the insect and the economic feasibility of the field treatments, considering that the cost of four anti-symbiont applications is similar to that of two insecticide treatments.

When evaluating the effect of stink bug feeding activity on hazelnuts, a positive effect on hazelnuts from the plot treated with the anti-symbiont biocomplex was detected in 2022, similar to the plot treated with lambda-cyhalothrin in terms of damage. Considering that the reduction in damage was greater than the effect on *H. halys* population trend, the presence of additional effects could be suggested. For example, the same biocomplex used here for anti-symbiont treatments has previously been shown to deter oviposition in the olive fruit fly *Bactrocera oleae* (Rossi) (Diptera: Tephritidae) [44]; in the case of *H. halys*, feeding deterrence cannot be excluded. However, the effect of symbiont-targeted control was reduced in 2023, when the highest levels of both adults and nymphs of *H. halys* were also detected, and in 2024, when damage to hazelnuts was similar in the plot treated with the anti-symbiont biocomplex and in the untreated control, but lower in the plot treated with lambda-cyhalothrin. In general, the lower level of damage observed in kernels from insecticide-treated plots agrees with the lower population of *H. halys* in that part of the field and with previous work [42]. The variable environmental conditions of each year (mainly in terms of temperature and precipitation [40]) may be involved in the lack of a consistent response throughout the years for the biocomplex treatment compared to the insecticide. In particular, the stink bug-associated damage was lower during 2022 compared to the other years, no matter the treatment, confirming that the intrinsic variability of each year can affect the efficacy of the treatment. Also, that year, the abundance of *H. halys* in field was lower (probably related to higher mean temperatures), which may suggest a higher efficacy of the symbiotic control under relatively lower infestation levels.

On the whole, our three-year field assessment of a symbiont-targeted control strategy against *H. halys* in a hazelnut orchard indicates that the interruption of symbiont acquisition by nymphs, although less effective than pyrethroid treatments in controlling adult *H. halys*, mostly reduced the damaged kernels compared to the control treatment. Additionally, we demonstrate that this approach does not attempt against the co-occurring entomofauna. In a context of increasing restrictions on the use of pesticides, the symbiotic control may provide an alternative mean that can be combined with other strategies such as biological control; moreover, it is not expected to induce pest resurgence phenomena. Future studies should focus on ameliorating the efficacy of field application by addressing operational aspects (e.g., application technique, number and frequency of applications) to achieve a more consistent reduction in yield losses.

## Figures and Tables

**Table 1:** Dates of treatments and surveys per year.

			Field Trial (Year)		
			2022	2023	2024
Application date	Treatment	Anti-symbiont Biocomplex	-	22-June	27-June
			29-June	30-June	4-July
			9-July	10-July	15-July
			19-July	19-July	24-July
		Lambda-cyhalothrin	22-June	23-June	26-June
			13-July	14-July	14-July
Survey date		Survey 0 (pre-treatment)	21-June	21-June	21-June
		Survey 1	26-June	26-June	28-June
		Survey 2	1-July	7-July	8-July
		Survey 3	14-July	12-July	19-July
		Survey 4	25-July	24-July	26-July

## Data Availability

The original contributions presented in this study are included in the article/supplementary material. Further inquiries can be directed to the corresponding author.

## Supplementary Materials

The following supporting information can be downloaded at: https://www.mdpi.com/article/10.3390/insects16070688/s1.

## References

[1] Paganizza V., Alabrese M., Brunori M., Rolandi S., Saba A. (2017). Insects in Agriculture: Traditional Roles and Beyond. Agricultural Law: Current Issues from a Global Perspective.

[2] Schowalter T.D., Noriega J.A., Tscharntke T. (2018). Insect Effects on Ecosystem Services—Introduction. Basic Appl. Ecol..

[3] FAOSTAT. https://www.fao.org/faostat/en/#data/QCL.

[4] Viggiani G. (1994). Current Management of Hazelnut Diseases and Pests. Acta Hortic..

[5] Chediack A., Liesch P.J., Shanovich H.N., Aukema B.H. (2022). Arthropod Community in Hybrid Hazelnut Plantings in the Midwestern United States. J. Insect Sci..

[6] AliNiazee M.T. (2001). Hazelnut Production without the Use of Broad Spectrum Disruptive Insecticides: Theory and Practice. Acta Hortic..

[7] Murray K., Jepson P. (2018). Integrated Pest Management Strategic Plan for Hazelnuts in Oregon and Washington.

[8] Dioli P., Leo P., Maistrello L. (2016). Prime segnalazioni in Spagna e in Sardegna della specie aliena *Halyomorpha halys* (Stål, 1855) e note sulla sua distribuzione in Europa (Hemiptera, Pentatomidae). Rev. Gaditana Entomol..

[9] Maistrello L., Vaccari G., Caruso S., Costi E., Bortolini S., Macavei L., Foca G., Ulrici A., Bortolotti P.P., Nannini R. (2017). Monitoring of the Invasive *Halyomorpha halys*, a New Key Pest of Fruit Orchards in Northern Italy. J. Pest. Sci..

[10] Faúndez E.I., Rider D.A. (2017). The Brown Marmorated Stink Bug *Halyomorpha halys* (Stål, 1855) (Heteroptera: Pentatomidae) in Chile. Arq. Entomoloxicos.

[11] Van der Heyden T., Saci A., Dioli P. (2021). First record of the brown marmorated stink bug *Halyomorpha halys* (Stål, 1855) in Algeria and its presence in North Africa (Heteroptera: Pentatomidae). Rev. Gaditana Entomol..

[12] Avila G.A., Seehausen M.L., Lesieur V., Chhagan A., Caron V., Down R.E., Audsley N., Collatz J., Bukovinszki T., Sabbatini Peverieri G. (2023). Guidelines and Framework to Assess the Feasibility of Starting Pre-Emptive Risk Assessment of Classical Biological Control Agents. Biol. Control.

[13] Bosco L., Moraglio S.T., Tavella L. (2018). *Halyomorpha halys*, a Serious Threat for Hazelnut in Newly Invaded Areas. J. Pest. Sci..

[14] Vaccino P., Guidone L., Corbellini M., Tavella L. (2008). Detection of Damage Due to Bug Feeding on Hazelnut and Wheat by Biochemical Techniques. Bull. Insectol..

[15] Kuhar T.P., Kamminga K. (2017). Review of the Chemical Control Research on *Halyomorpha halys* in the USA. J. Pest. Sci..

[16] Marchand P.A. (2023). Evolution of Plant Protection Active Substances in Europe: The Disappearance of Chemicals in Favour of Biocontrol Agents. Environ. Sci. Pollut. Res..

[17] Dara S.K. (2019). The New Integrated Pest Management Paradigm for the Modern Age. J. Integr. Pest. Manag..

[18] Moraglio S.T., Tortorici F., Visentin S., Pansa M.G., Tavella L. (2021). *Trissolcus Kozlovi* in North Italy: Host Specificity and Augmentative Releases Against *Halyomorpha halys* in Hazelnut Orchards. Insects.

[19] Tortorici F., Bombi P., Loru L., Mele A., Moraglio S.T., Scaccini D., Pozzebon A., Pantaleoni R.A., Tavella L. (2023). *Halyomorpha halys* and Its Egg Parasitoids *Trissolcus japonicus* and *T. mitsukurii*: The Geographic Dimension of the Interaction. NeoBiota.

[20] Yang Z.-Q., Yao Y.-X., Qiu L.-F., Li Z.-X. (2009). A New Species of *Trissolcus* (Hymenoptera: Scelionidae) Parasitizing Eggs of *Halyomorpha halys* (Heteroptera: Pentatomidae) in China with Comments on Its Biology. Ann. Entomol. Soc..

[21] Qadri M., Short S., Gast K., Hernandez J., Wong A.C.-N. (2020). Microbiome Innovation in Agriculture: Development of Microbial Based Tools for Insect Pest Management. Front. Sustain. Food Syst..

[22] Duron O., Noël V. (2016). A Wide Diversity of *Pantoea* Lineages Are Engaged in Mutualistic Symbiosis and Cospeciation Processes with Stinkbugs. Environ. Microbiol. Rep..

[23] Prado S.S., Rubinoff D., Almeida R.P.P. (2006). Vertical Transmission of a Pentatomid Caeca-Associated Symbiont. Ann. Entomol. Soc..

[24] Gonella E., Orrù B., Marasco R., Daffonchio D., Alma A. (2020). Disruption of Host-Symbiont Associations for the Symbiotic Control and Management of Pentatomid Agricultural Pests—A Review. Front. Microbiol..

[25] Checchia I., Andreolli M., Lanza F., Santoiemma G., Mori N., Pasini M., Lampis S., Felis G.E. (2025). Testing Low-Risk Bioactive Compounds on *Halyomorpha halys*: An Improved Pipeline of Analyses to Investigate Their Effects on the Bacterial Endosymbiont *Candidatus* Pantoea Carbekii. Pest. Manag. Sci..

[26] Gonella E., Orrù B., Alma A. (2019). Egg Masses Treatment with Micronutrient Fertilizers Has a Suppressive Effect on Newly-Emerged Nymphs of the Brown Marmorated Stink Bug *Halyomorpha halys*. Entomol. Gen..

[27] Gonella E., Orrù B., Alma A. (2022). Symbiotic Control of *Halyomorpha halys* Using a Microbial Biopesticide. Entomol. Gen..

[28] Taylor C.M., Johnson V., Dively G. (2017). Assessing the Use of Antimicrobials to Sterilize Brown Marmorated Stink Bug Egg Masses and Prevent Symbiont Acquisition. J. Pest. Sci..

[29] Gonella E., Alma A. (2023). The Role of Symbiont-Targeted Strategies in the Management of Pentatomidae and Tephritidae Pests under an Integrated Vision. Agronomy.

[30] Dho M., Gonella E., Alma A. (2025). Field Evaluation of Symbiont-Targeted Control of *Halyomorpha halys* in Hazelnut Crop. Crop Prot..

[31] Prieto S.V., Brunetti M., Magoga G., Orrù B., Gonella E., Montagna M., Alma A. (2023). The Intrinsic Diversity of *Nezara viridula* Gut Symbionts Affects the Host Fitness Decline Promoted by Primary Symbiont Elimination. Entomol. Gen..

[32] Rider D.A., Schwertner C.F., Vilímová J., Rédei D., Kment P., Thomas D.B. (2018). Higher Systematics of the Pentatomoidea. Invasive Stink Bugs and Related Species (Pentatomoidea).

[33] Memoli A., Albanese D., Esti M., Lombardelli C., Crescitelli A., Di Matteo M., Benucci I. (2017). Effect of Bug Damage and Mold Contamination on Fatty Acids and Sterols of Hazelnut Oil. Eur. Food Res. Technol..

[34] Chagnon M., Kreutzweiser D., Mitchell E.A.D., Morrissey C.A., Noome D.A., Van der Sluijs J.P. (2015). Risks of Large-Scale Use of Systemic Insecticides to Ecosystem Functioning and Services. Environ. Sci. Pollut. Res..

[35] Martelli F., Batterham P. (2025). Agrochemicals: Insect Declines in a Warming World. Curr. Biol..

[36] Sonoda S., Izumi Y., Kohara Y., Koshiyama Y., Yoshida H. (2011). Effects of Pesticide Practices on Insect Biodiversity in Peach Orchards. Appl. Entomol. Zool..

[37] Gantner M., Jaśkiewicz B. (2002). Beetles (Coleoptera) Occurring on Hazel (*Corylus* L.) in Different Habitat Conditions. Hortorum Cultus.

[38] Obrycki J.J., Kring T.J. (1998). Predaceous Coccinellidae in Biological Control. Annu. Rev. Entomol..

[39] Orrù B., Moraglio S.T., Tortorici F., Gonella E., Tavella L., Alma A. (2023). No Adverse Effects of Symbiotic Control on the Parasitism of *Halyomorpha halys* by Egg Parasitoids. J. Pest. Sci..

[40] Agenzia Regionale per la Protezione Ambientale del Piemonte. https://www.arpa.piemonte.it/.

[41] Fisher J.J., Rijal J.P., Zalom F.G. (2021). Temperature and Humidity Interact to Influence Brown Marmorated Stink Bug (Hemiptera: Pentatomidae) Survival. Environ. Entomol..

[42] De Benedetta F., Giaccone M., Pica F., Lisanti M.T., Vinale F., Turrà D., Giacca G.M., Bernardo U. (2023). Fruit Phenology of Two Hazelnut Cultivars and Incidence of Damage by *Halyomorpha halys* in Treated and Untreated Hazel Groves. Horticulturae.

[43] Rice K.B., Bergh C.J., Bergmann E.J., Biddinger D.J., Dieckhoff C., Dively G., Fraser H., Gariepy T., Hamilton G., Haye T. (2014). Biology, Ecology, and Management of Brown Marmorated Stink Bug (Hemiptera: Pentatomidae). J. Integr. Pest. Manag..

[44] Checchia I., Perin C., Mori N., Mazzon L. (2022). Oviposition Deterrent Activity of Fungicides and Low-Risk Substances for the Integrated Management of the Olive Fruit Fly *Bactrocera Oleae* (Diptera, Tephritidae). Insects.

